# CT Chest findings in IgG4-related disease

**DOI:** 10.1080/07853890.2025.2489745

**Published:** 2025-04-17

**Authors:** Ye Liu, Yongkang Nie

**Affiliations:** Department of Diagnostic Radiology, The First Medical Center of Chinese, PLA General Hospital

**Keywords:** IgG4 related diseases, IgG4 related lung diseases, multi-slice spiral CT, multi-slice spiral CT imaging manifestations

## Abstract

**Purpose:**

To investigate the multi-slice spiral Computed Tomography(MSCT) findings of chest involvement in IgG4-related diseases and to improve doctors’ understanding of this disease.

**Materials and Methods:**

We retrospectively analyzed the clinical and imaging data of 67 patients with clinically confirmed or suspected IgG4-related diseases.

**Results:**

Sixty patients (89.60%) had abnormal chest CT findings. Among them, 47 patients (70.10%) had enlarged mediastinal lymph nodes. Thickening of the tracheal and tracheobronchial perivascular walls was observed in 35 patients (52.20%). Nodules were observed in 29 patients (43.30%). Patches or ground-glass opacities were observed in 15 cases (22.40%); bilateral enlarged axillary lymph nodes, 9 (13.40%); bilateral enlarged hilar lymph nodes, 3 (4.50%); and, interstitial changes, 3 (11.90%). Pleural and pericardial effusion occurred in five cases (7.46%, two bilateral and three unilateral) and three cases (4.50%), respectively. Seven (10.45%) patients showed no obvious abnormalities. The abnormally elevated IgG4 level (>135 mg/dL) was positively correlated with thickening of the tracheal and tracheobronchial walls (*r* = 0.328, *p* = 0.007) and enlargement of mediastinal lymph nodes (r = −0.252, *p* = 0.039), and the logistic regression model 1 showed that the incidence of lung as the first symptom was higher in patients with bilateral enlarged hilar lymph nodes on chest images.

**Conclusion:**

Chest CT is of great significance for the diagnosis and follow-up of IgG4-RLD. Chest CT scans should be suggested to be performed when ocular symptoms, bilateral enlarged hilar lymph nodes, pancreatitis, pituitary adenitis, Takayasu arteritis, or abnormally elevated IgG4 levels (>135 mg/dL) are present, and IgG4-RLD should be considered.

IgG4-related disease (IgG4-RD) is an immune-mediated disease that can cause fibrous inflammation in almost any organ [1], with unknown etiology, and mostly in Asian studies. IgG4-RD is characterized by elevated serum IgG4 levels and extensive infiltration of IgG4+ plasma cells IgG4-RD involves multiple organs, including the lacrimal gland, salivary gland, lungs, pancreas, gallbladder, and kidneys, and it may also cause retroperitoneal fibrosis (RPF). Among these different lesions, up to 35% of IgG4-RD patients [2] have abnormal chest imaging findings, including pulmonary nodules, thickened bronchial vascular bundles, thickened bronchial walls, masses, consolidation, thickened pleura, pleural effusion, aortitis, sclerosing pericarditis, lymphadenopathy, and paravertebral masses. Other reports found chest involvement in approximately 14% of patients by chest imaging examination, and salivary gland and systemic involvement were more often observed in them [3,4]. Zen et al. [5,6] confirmed that IgG4-RD can occur in lung tissue in 2005, and they named it IgG4-related lung disease (IgG4-RLD) in 2009. At the International Symposium on IgG4-RD held in Boston in October 2011, it was recommended that IgG4-RD involving the lungs be named as IgG4-RLD [7]. Its epidemiology remains poorly described because of its relatively recent recognition as a discrete condition, yet the disease is now seen by both generalists and specialists all across the world.

Chest CT examination is of crucial importance for IgG4-RLD. It helps in disease discovery and preliminary diagnosis by identifying lesions and indicating the presence of the disease. It can accurately assess the scope and degree of the disease, including the involvement of the lungs and other chest structures. It is beneficial for disease differential diagnosis, distinguishing it from other lung diseases and dynamically observing the evolution of lesions. It is also an important mean for monitoring treatment effects, which can evaluate treatment responses and guide treatment adjustments. This article reviews the chest imaging and clinical laboratory findings of IgG4-RD to improve the understanding of this disease in imaging and clinical physicians.

## Methods and ethical concern

This study retrospectively analyzed the outpatient and inpatient patients who visited the Department of Rheumatology and Immunology, Hepatobiliary Surgery, Respiratory Medicine, Ophthalmology, Gastroenterology, Neurology, Otorhinolaryngology and Nephrology of the First Medical Center of the Chinese People’s Liberation Army General Hospital from 2012 to 2021. Among 1,539 suspected patients, there were 541 patients who were diagnosed, highly suspected or suspected. Among them, 67 patients were diagnosed or highly suspected patients and underwent chest CT examinations.

**Figure 1.**
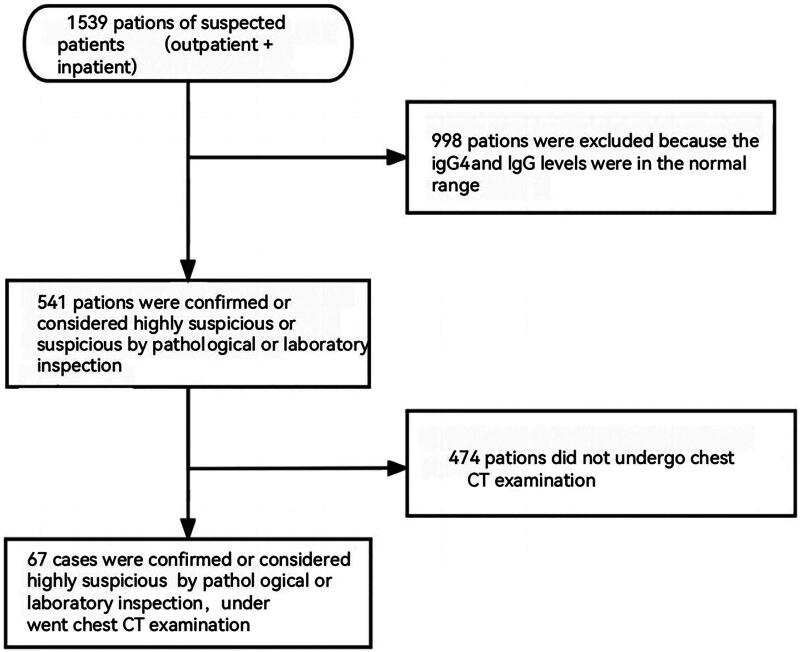
Inclusion flow of IgG4-RLD patients. From 1 January 2012 to 31 December 2021, 1539 suspected outpatients and inpatients who visited various departments of the First Medical Center of the Chinese People’s Liberation Army General Hospital were collected. Among them, 998 patients were excluded because the laboratory tests of IgG and IgG4 were within the normal range. 541 patients were confirmed diagnosed, highly suspected, or suspected through pathological or laboratory tests. 474 patients were excluded as they did not undergo chest CT examinations. The remaining 67 patients were collected and enrolled in the study.

Clinical data:Among 1,539 suspected patients, there were 541 patients who were diagnosed, highly suspected or suspected. Among them, 67 patients were diagnosed or highly suspected patients and underwent chest CT examinations, and including 54 cases of plain chest CT scans and 13cases of plain and enhanced chest CT scans. We analyzed the patients’ clinical, serological, and imaging characteristics and treatment responses (Figure 1 Inclusion flow of IgG4-RLD patients). There were 45 men and 22 women (M/F ratio, 23:11), and the average age was 59.63 ± 10.7 years (range, 25–80 years). The clinical manifestations were diverse, and almost half of the patients were initially diagnosed with IgG4-RD in the rheumatology department. Clinical symptoms may appear in the chest or not, and the symptoms include cough, shortness of breath, chest pain, and asthma. The average duration of symptoms before diagnosis was 20 months (range, 1–204 months). Nearly half (25/67) of the patients had a history of smoking. The exclusion criteria were as follows [1]: presence of other lung diseases, including lung cancer, lung infection, and interstitial lung disease [2], other diseases (such as Sjögren syndrome) that can cause lung involvement similar to that of IgG4-RD, and [3] patients with missing chest CT images or those who could not be reexamined.

The chest findings of IgG4-RD include mediastinal lymphadenopathy, fibrotic mediastinitis, pleural lesions, airway or pulmonary parenchymatous diseases, and pericardial lesions, which were confirmed by clinical manifestations, serological examinations, and imaging or pathological findings.

## Diagnosis of IgG4-RD

IgG4-RD is an autoimmune disease with multi-organ involvement and various clinical manifestations. Its diagnosis is a comprehensive result based on clinical, serological, radiological, and histopathological findings. None of these findings provided clear evidence for a diagnosis. Cross-sectional imaging (ultrasound, CT, and Magnetic Resonance ImagingMRI) is an important component of the diagnosis and treatment of IgG4-RD. Patients were classified according to the 2011comprehensive diagnostic criteria for IgG4-RD and the 2019 classification criteria of the American College of Rheumatology (ACR)/European League Against Rheumatism (EULAR) (score 20) [8,9]. Data regarding age, sex, time of diagnosis, organ involvement, biological assessment, treatment, and outcomes were collected.

Chest CT examinations of confirmed and suspected patients were performed according to the diagnostic criteria for IgG4-RLD reported by Matsui et al. [10,11] (Table 1 Summary of diagnostic criteria for IgG4-RRD). Abnormalities were described according to the Fleischner vocabulary [12] as follows: bronchiolitis (tree-in-bud pattern or alveolar nodules), bronchiectasis, solid nodules, ground-glass opacity (GGO), consolidation, pleural effusion or pleural thickening, peribronchovascular thickening, mediastinal lymph nodes, bronchial mucoid impaction, interlobular septal thickening, honeycomb and grid-like changes, structural deformation, and mediastinal fibrosis (paravertebral soft-tissue zone).

**Table 1.** Summary Of diagnostic criteria for IgG4-RRD.

1. Abnormal shadow on chest CT
Hilar/mediastinal lymphadenopathy
Thickening of the bronchial wall, bronchovascular bundle, and interlobular septal wall
Nodular shadows, infiltrative shadows, pleural thickening/effusion
2. Elevated serum IgG4 levels (≥135 mg/dl)
3. Pathological findings satisfying ≥2 from the following: (a: ≥3 items, b: ≥ 2 items)
1) Dense lymphoplasmacytic cell infiltration into respiratory organ tissues
2) IgG4^+^/IgG^+^ cell ratio >40% and/or >10 IgG4^+^ cells/high-power field
3) Obliterative phlebitis or arteritis
4) Characteristic fibrosis, typically a storiform pattern
4. Presence of lesions in the extrathoracic organs satisfying the diagnostic criteria for IgG4-related diseases.
(Reference findings) Hypocomplementemia.
Definite diagnosis (definite):1 + 2 + 3a, 1 + 2 + 3b + 4
Probable diagnosis (probable):1 + 2 + 4,1 + 2 + 3b + reference finding.
Possible diagnosis (possible):1 + 2 + 3b

Definite diagnosis (definite):1 + 2 + 3a, 1 + 2 + 3b + 4.

Probable diagnosis (probable):1 + 2 + 4,1 + 2 + 3b + reference finding.

Possible diagnosis (possible):1 + 2 + 3b.

### Inspection methods

All 67 patients underwent plain chest scans or plain and enhanced chest scans using Philips Brilliance 256 iCT (Netherlands) or Siemens Sensation Cardiac (Germany). The patients were placed in the supine position with their arms raised. The scan was performed in a head-advanced manner, from the apex of the lung to 3 cm below the diaphragm. The whole-chest CT scan was completed when the patient held his breath at the end of the inhalation. The scanning parameters were as follows: tube voltage, 120 kVp; tube current modulation, automatic; and, reconstructive thickness, 1.0–1.5 mm. The lung window had a width of 1600HU, and the window position was -600HU. The mediastinal window had a width of 400HU, and the window position was 40HU. Contrast-enhanced scanning was performed with 70–90 ml of non-ionic contrast medium (iohexol or iopromide, 300 mg I/mL) at a flow rate of 3.5 ml/s. Arterial and venous phase scans were performed 25–30s and 60–65s after injection of the contrast medium, respectively. The re-examination conditions were consistent with those of the first scan. After analysis, the images were sent to a picture archiving and communication system. The images remained in their original size and were displayed randomly. The location, size, and shape of the lesions; lung interface; edge of the lesion; length of burr; relationship of the lesion with the bronchus, blood vessels, and pleura; lesion composition; calcification; surrounding status; pleural effusion; and, hilar and mediastinal lymph node enlargement were evaluated by two experienced senior radiologists using a double-blind method. When opinions differed, a consensus was reached through consultation.

### Statistics

SPSS (V24.0) and R (4.0.2) were used for statistical analyses. Quantitative data with normal and non-normal distribution were described as mean ± standard deviation (SD) and median and quartile spacing (IQRs), respectively, and qualitative data were described as percentages. Spearman correlation analysis was used to analyze the correlation of variables that did not follow a normal distribution, that is, between pulmonary imaging abnormalities, initial symptoms, and seroimmunity abnormalities. Logistic regression was used to explore the abnormal immune indices and lung imaging changes that affected the first symptoms of the lungs. Statistical significance was set at *p* < 0.05.

This study was approved by the Medical Ethics Committee of the First Medical Center of the PLA General Hospital and conformed to the principles outlined in the Declaration of Helsinki. I obtained verbal informed consent from the individuals participating in the study and kept their details confidential. Because this study was retrospective, the authors obtained verbal informed consent by telephone follow-up, and verbal informed consents were approved by the Medical Ethics Committee of the First Medical Center of PLA General Hospital.

## Results

### Patient characteristics

Among 67 patients, 62 patients are pathologically confirmed, and 5 patients are highly suspected. The first affected organ was the pancreas in 28 patients (41.8%), followed by the eyes, bile ducts, and lungs (9 cases, 13.4% for each organ); retroperitoneum (8 cases, 11.9%); lymph nodes (5 cases, 7.5%); blood vessels (4 cases, 6.00%); and, meninges, connective tissue, nasopharynx, pituitary, and kidney (2 cases, 3.00%, for each organ).

Serum immune indices showed abnormally elevated levels of IgE (>100IU/L) in 26 patients (39.40%); IgG (>1660 g/L), 39 (58.20%); IgG4 (>135 mg/dL), 62 (92.50%); and, C-reactive protein (CRP) (>5 mg/L), 8 (11.90%).

**Figure 2.**
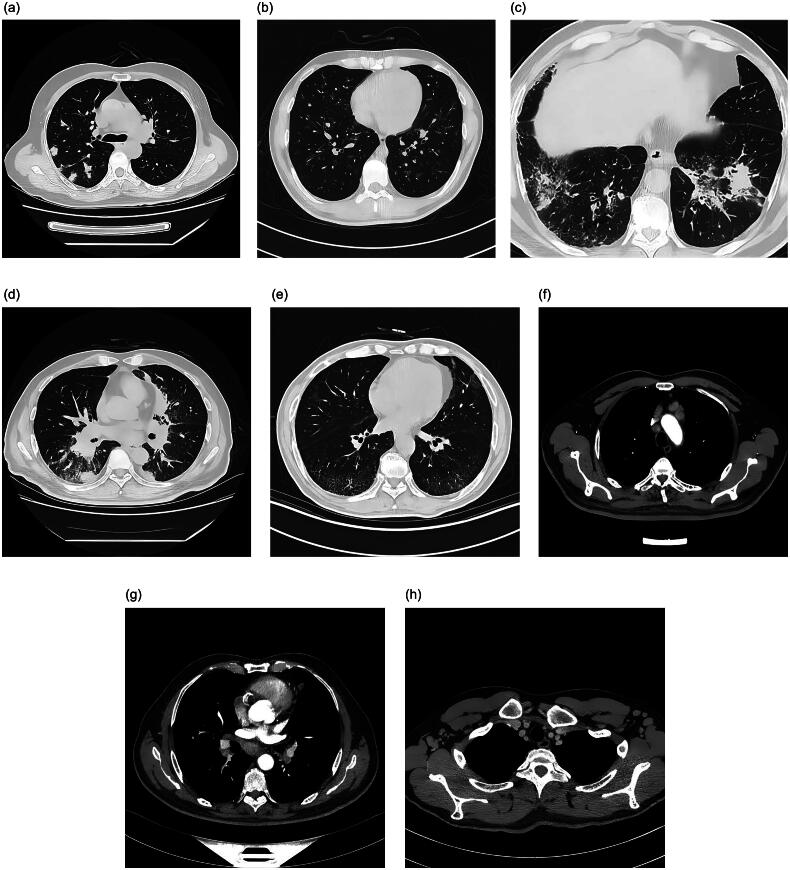
Abnormal imaging findings on chest CT. Chest abnormalities in patients with IgG4-related diseases (each computed tomography [CT] scan section is of a different patient). Axial CT scan of the lung window (a)–(e): (a) shows multiple nodules of different sizes in both lungs, (b) Thickening of bronchiolar walls in both lungs. (c) Consolidation in both lungs along bronchi. (d) Right paraphenous mass. (e) Small-grid and ground-glass opacities around the lungs, indicating interstitial changes. Mediastinal window axial enhanced CT (f)–(h): Multiple enlarged lymph nodes in the mediastinum, bilateral hila, and bilateral axillary regions.

Chest imaging revealed that 60 patients (89.60%) had abnormal lungs. Among them, 47 (70.10%) patients had enlarged mediastinal lymph nodes. Thickening of the tracheal and tracheobronchial perivascular walls was observed in 35 patients (52.20%), including the tree-in-bud sign in 13 patients. Bronchiectasis was observed in nine cases; thickening of the tracheobronchial wall, 26; consolidation or mucoid impaction, 1; and, nodules, 29 (43.30%). The nodules were solid, approximately 5–15 mm in size, with smooth edges and no obvious burrs. Patches or ground-glass opacities were observed in 15 cases (22.40%); bilateral enlarged axillary lymph nodes, 9 (13.40%); bilateral enlarged hilar lymph nodes, 3 (4.50%); and, interstitial changes, 8 (11.90%) (Table 2 Patient characteristics, Table 3 Imaging indexes of lungs and Figure 2 Abnormal imaging findings on chest CT). Bilateral pleural effusion occurred in two cases (3.00%); unilateral pleural effusion, 3 (4.50%); and, pericardial effusion, 3 (4.50%). Seven (10.45%) patients showed no obvious abnormalities. Peribron­chovascular involvement (52.20%) and lymph node enlargement (70.10%) were the most common chest abnormalities, followed by nodules (43.30%), patches or GGO (22.40%), and interstitial changes (11.90%). Fifty-three patients (79.10%) had at least two chest abnormalities (Table 4 Logistic regression analysis of the probability of lung involvement).

**Table 2.** Patient characteristics.

	Mean ± SD/N(%)
Gender(M, %)	45, 67.20%
Age X ± SD	59.63 ± 10.7
Smoking (Smokers, %)	25, 37.30%
First symptom	
Eyelid swelling, %	9, 13.40%
Diffuse thickening of the vessel wall, %	4, 6.00%
Autoimmune pancreatitis, %	28, 41.80%
hypertrophic pachymeningitis, %	2, 3.00%
Sclerosing cholangitis, %	9, 13.40%
Connective tissue disease, %	2, 3.00%
Bronchiolitis and interstitial pneumonia, %	9, 13.40%
Retroperitoneal fibrosis, %	8, 11.90%
A mass in the nasopharynx, %	2, 3.00%
Diffuse enlargement of the pituitary gland, %	2, 3.00%
Lymph node enlargement, %	5, 7.50%
Nephritis, %	2, 3.00%
Biochemistry index	
Abnormal IgE, %	26, 39.40%
Abnormal IgG, %	39, 58.20%
Abnormal IgG4, %	62, 92.50%
Abnormal Crp, %	8, 11.90%

The incidence of gender, age, smoking status, first symptoms of the disease, and abnormal laboratory tests (IgE, IgG, IgG4, and CRP) in 67 patients.

**Table 3.** Imaging indexes of lungs.

	Mean ± SD/N(%)
Lung imaging (Abnormal, %)	60, 89.60%
Nodules, %	29, 43.30%
Patches or GGO, %	15, 22.40%
Trachea and bronchus wall thickening, %	35, 52.20%
Interstitial changes, %	8, 11.90%
Enlarged mediastinal lymph nodes, %	47, 70.10%
Enlarged bilateral hilar nodes, %	3, 4.50%
Enlarged bilateral axillary lymph nodes, %	9, 13.40%
Bilateral pleural effusion, %	2, 3.00%
Right pleural effusion, %	3, 4.50%
Pericardial effusion, %	3, 4.50%

The incidence of abnormal pulmonary imaging findings in 67 patients. The abnormal imaging findings include nodules, patchy or ground – glass opacities, interstitial changes, mediastinal, bilateral hilar, bilateral axillary enlarged lymph nodes, bilateral pleural effusions, right – sided pleural effusion, and pericardial effusion.

**Table 4.** Logistic regression analysis of the probability of lung involvement.

	Normal		Nodules		Patch or GGO		Thickening of trachea and bronchial tube walls		interstitial change		Mediastinal lymph nodes enlargement		Bilateral hilar lymph nodes enlargement		Bilateral axillary lymph nodes enlargement		Bilateral pleural effusion		Right pleural effusion		pericardial effusion	
	r	*ρ*	r	*ρ*	r	*ρ*	r	*ρ*	r	*ρ*	r	*ρ*	r	*ρ*	r	*ρ*	r	*ρ*	r	*ρ*	r	*ρ*
IgE > 100iul	0.024	0.846	0.098	0.432	−0.141	0.258	0.224	0.071	−0.014	0.909	0.127	0.31	−0.027	0.829	0.081	0.52	0.038	0.76	−0.176	0.158	0.122	0.33
IgG > 1660g	−0.007	0.953	0.068	0.583	0.02	0.876	0.22	0.074	0.219	0.075	0.042	0.733	0.037	0.765	−0.021	0.865	−0.029	0.815	−0.256*	0.037	0.183	0.137
igg4 > 135mgdl	−0.235	0.056	0.063	0.613	−0.082	0.508	0.328**	0.007	0.115	0.352	0.252*	0.039	0.068	0.585	0.124	0.319	0.055	0.658	−0.185	0.134	0.068	0.585
crp > 5mgl	−0.126	0.311	−0.043	0.73	−0.087	0.482	−0.017	0.895	−0.136	0.274	0.039	0.754	−0.08	0.521	−0.01	0.936	0.206	0.095	0.143	0.249	−0.08	0.521

Logistic regression model 1 showed that, in patients with bilateral enlarged hilar lymph nodes on chest images, the probability of the lung as the first symptom increased (OR = 16.000, 95% CI: 1.280–200.010), and after adjusting for sex, age, and smoking factors, logistic regression model 2 showed the same trend (OR = 19.694, 95% CI: 1.148–337.726).

### Correlation between chest imaging, the first-involved organs, and symptoms

The correlation analysis showed that there were positive correlations between ocular symptoms and normal lung imaging results (r = −0.295, *p* = 0.015), lung symptoms and bilateral enlarged hilar lymph nodes (*r* = 0.338, *p* = 0.005), lymph node symptoms and bilateral axillary lymph node enlargement (*r* = 0.388, *p* = 0.001), pituitary symptoms and right pleural effusion (*r* = 0.386, *p* = 0.001), vascular symptoms and right pleural effusion (*r* = 0.250, *p* = 0.041), and pancreatic symptoms and pericardial effusion (*r* = 0.256, *p* = 0.037) (Table 5 Correlation between lung imaging finding and first symptoms).

**Table 5.** Correlation between lung imaging finding and first symptoms.

	Model 1		Model 2	
	OR value (95%)	*P* value	OR value (95%)	*P* value
N	1		1	
Y	16.000 (1.280–200.010)	0.031	19.694 (1.148–337.726)	0.04

The correlation analysis showed that there were positive correlations between ocular symptoms and normal lung imaging results (r = –0.295, *ρ* = 0.015), lung symptoms and bilateral enlarged hilar lymph nodes (*r* = 0.338, *ρ* = 0.005), lymph node symptoms and bilateral axillary lymph node enlargement (*r* = 0.388, *ρ* = 0.001), pituitary symptoms and right pleural effusion (*r* = 0.386, *ρ* = 0.001), vascular symptoms and right pleural effusion (*r* = 0.250, *ρ* = 0.041), and pancreatic symptoms and pericardial effusion (*r* = 0.256, *ρ* = 0.037).

### Correlation between lung imaging findings and abnormal serum immune indices

The correlation analysis showed that abnormally elevated IgG levels (>1660 g/L) were negatively correlated with right pleural effusion (r = −0.256, *p* = 0.037), and abnormally elevated IgG4 levels (>135 mg/dL) were positively correlated with a thickened tracheobronchial wall (*r* = 0.328, *p* = 0.007) and enlarged mediastinal lymph nodes (r = −0.252, *p* = 0.039) (Table 6 Correlation between lung imaging findings and abnormal serum immune indices).

**Table 6.** Correlation between lung imaging findings and abnormal serum immune indices.

	Normal		Nodules		Patch or GGO		Thickening of trachea and bronchial tube walls		interstitial change		Mediastinal lymph nodes enlargement		Bilateral hilar lymph nodes enlargement		Bilateral axillary lymph nodes enlargement		Bilateral pleural effusion		Right pleural effusion		pericardial effusion	
	r	*ρ*	r	*ρ*	r	*ρ*	r	*ρ*	r	*ρ*	r	*ρ*	r	*ρ*	r	*ρ*	r	*ρ*	r	*ρ*	r	*ρ*
Eye	0.295*	0.015	0.009	0.941	−0.107	0.391	−0.061	0.621	−0.145	0.242	−0.03	0.81	−0.085	0.493	−0.155	0.21	0.188	0.127	−0.085	0.493	−0.085	0.493
Vascular	−0.086	0.489	0.034	0.784	−0.135	0.275	−0.011	0.928	−0.093	0.455	0.027	0.83	−0.055	0.661	0.085	0.492	−0.044	0.722	0.250*	0.041	−0.055	0.661
Pancreas	0.007	0.953	0.115	0.355	−0.02	0.876	0.023	0.856	0.155	0.212	0.024	0.849	−0.037	0.765	0.11	0.376	0.029	0.815	−0.183	0.137	0.256*	0.037
Meninges	0.227	0.065	−0.153	0.216	−0.094	0.448	−0.183	0.137	−0.065	0.604	−0.077	0.534	−0.038	0.76	0.188	0.127	−0.031	0.805	−0.038	0.76	−0.038	0.76
Bile duct	−0.135	0.278	−0.079	0.525	0.103	0.405	0.026	0.834	0.125	0.314	−0.03	0.81	−0.085	0.493	−0.155	0.21	−0.069	0.578	0.126	0.308	−0.085	0.493
Connective tissue	−0.06	0.63	0.024	0.849	0.116	0.349	0.168	0.175	−0.065	0.604	0.114	0.357	−0.038	0.76	−0.069	0.578	−0.031	0.805	−0.038	0.76	−0.038	0.76
Lung	−0.135	0.278	0.009	0.941	−0.002	0.99	0.201	0.102	−0.01	0.936	0.066	0.598	0.338**	0.005	−0.027	0.829	−0.069	0.578	−0.085	0.493	−0.085	0.493
Retroperitoneum	0.025	0.843	0.05	0.688	0.023	0.853	0.076	0.543	0.148	0.231	−0.062	0.621	−0.08	0.521	−0.145	0.242	0.206	0.095	−0.08	0.521	−0.08	0.521
nasopharynx	0.227	0.065	−0.153	0.216	−0.094	0.448	−0.008	0.95	−0.065	0.604	−0.077	0.534	−0.038	0.76	−0.069	0.578	−0.031	0.805	−0.038	0.76	−0.038	0.76
Pituitary gland	−0.06	0.63	0.024	0.849	0.116	0.349	−0.183	0.137	−0.065	0.604	−0.077	0.534	−0.038	0.76	−0.069	0.578	−0.031	0.805	0.386**	0.001	−0.038	0.76
Lymphonodus	−0.097	0.435	−0.133	0.282	−0.153	0.218	0.044	0.723	−0.105	0.4	−0.063	0.613	−0.061	0.621	0.388**	0.001	−0.05	0.689	−0.061	0.621	−0.061	0.621
Kidney	−0.06	0.63	0.024	0.849	−0.094	0.448	0.168	0.175	0.206	0.095	0.114	0.357	−0.038	0.76	−0.069	0.578	−0.031	0.805	−0.038	0.76	−0.038	0.76

The correlation analysis showed that abnormally elevated IgG levels (>1660 g/L) were negatively correlated with right pleural effusion (r = –0.256, *ρ* = 0.037), and abnormally elevated IgG4 levels (>135 mg/dL) were positively correlated with a thickened tracheobronchial wall (*r* = 0.328, *ρ* = 0.007) and enlarged mediastinal lymph nodes (r = –0.252, *ρ* = 0.039).

### Main indicators affecting initial chest symptoms

Sex, age, smoking status, serum immune-related indices, and chest imaging findings were included in the logistic regression model, and the variables in the model were screened using the forward stepwise regression method. The results showed that only bilateral enlarged hilar lymph nodes were included in Model 1. Logistic regression model 1 showed that, in patients with bilateral enlarged hilar lymph nodes on chest images, the probability of the lung as the first symptom increased (OR = 16.000, 95% CI: 1.280–200.010), and after adjusting for sex, age, and smoking factors, logistic regression model 2 showed the same trend (OR = 19.694, 95% CI: 1.148–337.726) (Table 4 Logistic regression analysis of the probability of lung involvement).

## Discussion

Sixty patients (89.55%) had abnormal chest images, showing one or more types of abnormal chest images, which is consistent with the literature. Approximately 17.6–40.0% of patients with IgG4-RD had lung involvement, whereas approximately 37.5–87.5% of patients with IgG4-RLD had extrapulmonary or extrathoracic lesions [9,13–17]. IgG4-RLD usually coexists with other systemic diseases not involving the chest or lungs; however, only a few cases involve the lungs alone. Therefore, patients with IgG4-RLD whose lung lesions are the first symptom or the lung is the single lesion site should be followed-up regularly, as other organs outside the lungs may be involved in the later stages of the disease. The clinical and imaging manifestations of IgG4-RLD are nonspecific, and the symptoms, including cough, chest pain, dyspnea, and hemoptysis, depend on the lesion site. In addition, there may be low fever and weight loss, and approximately half of the patients have no respiratory symptoms [13–17]. In this study, nine (13.43%) patients were admitted because of lung symptoms, and the main symptoms were cough, expectoration, chest pain, asthma, and dyspnea; 58 (86.57%) patients had other organs as the first-involved organ, and all patients had ≥2 organs involved at the time of diagnosis. Scholars worldwide have found that middle-aged and older men are slightly more common; the incidence ratio in men to women is 2.6:1, and the average age of onset is 58 years. This finding is consistent with the results of the present study [17].

The most common lesions in the 67 patients were peribronchovascular involvement (52.20%) and lymph node enlargement (70.10%). Regarding the main chest manifestations, bronchovascular lesions (56%) and lymph node enlargement (31%) were the most common [10], followed by nodules (43.30%) and patches or GGO (22.40%), whereas interstitial changes (11.90%) were rare. Lv et al. [18] reported that pleural effusion may be unilateral or bilateral, with or without pericardial effusion. There were three cases of unilateral pleural effusion; two cases, bilateral pleural effusion; and, two, pericardial effusion. The incidence of pleural effusion, pleural thickening, and mediastinal fibrosis was lower than those of the other lung lesions. Fifty-three patients (79.10%) had at least two chest abnormalities. No priority correlation was found among these abnormal manifestations. There were no significant differences in sex, age, IgG4 levels, or smoking status. Abnormally high IgG4 levels (>135 mg/dL) were positively correlated with tracheobronchial wall thickening (*r* = 0.328, *p* = 0.007) and mediastinal lymph node enlargement (r = −0.252, *p* = 0.039).

Some abnormal manifestations are associated with specific extrathoracic organ manifestations, such as GGO with pancreatitis, peribronchovascular involvement with nephritis, interstitial diseases with eosinophilia, and lymph node enlargement with salivary adenitis. Although this association, found in limited cases, is finite, it can reflect the changes observed in the pathological changes between organs [19]. This relevance is also reflected in this study.

The differential diagnoses of IgG4-RLD includes sarcoidosis [20], Anti-Neutrophil Cytoplasmic Antibodies(ANCA)-associated vasculitis, connective tissue disease-associated interstitial pneumonia [21], idiopathic interstitial pneumonia (IIP), lymphoma [22,23], primary or metastatic lung cancer, Erdheim–Chester disease [24], infection (including bacterial infection and non-mycobacterial or fungal infection), bronchial asthma, Castleman disease, and myofibroblastic tumor [25]. To exclude these diagnoses, new biopsies, pathological tissue reviews, or additional laboratory tests may be helpful.

Compared with sarcoidosis, hilar and mediastinal lymph node enlargement is relatively less prominent, and is mostly mild and asymmetrical. Common CT manifestations of ANCA – associated vasculitis include ground – glass opacities, which mostly suggest alveolar hemorrhage or interstitial inflammation; nodular shadows may also be present, with nodules varying in size and having edges that can be either clear or blurred; there are also consolidation shadows, and bronchial air – filling signs can be seen in some of the consolidation shadows. In addition, cavity formation may occur, especially being more common in patients with Granulomatosis with polyangiitis. For infectious diseases, taking tuberculosis as an example, pulmonary tuberculosis can present various forms such as patchy shadows, nodular shadows, and cavity formation in the lungs. Pulmonary fungal infections may show nodules, consolidation, ground-glass opacities, etc. Sometimes, a ‘halo sign’ (ground-glass-like low-density shadow around the nodule) can be seen.

Our study suggests that IG4-RD cannot be solely diagnosed on radiological features and will require clinical, biochemical and histo-cytological evidence to support diagnosis. We suggest that thoracic CT scanning during the initial stage of the disease be carefully performed to identify peribronchovascular thickening and soft tissue-like band lesions, which can be considered more specific and helpful for diagnosis. IgG4-RD with multiple organ involvement is a risk factor for recurrence; thus, timely and effective identification of chest involvement helps determine the prognosis [26].

This study has some limitations. First, it was a single-center study; thus, there was bias in patient enrolment. Whether these results can be extended to other patients with different backgrounds remains unclear. Second, the number of patients undergoing lung biopsy was relatively small, and the tissues used for the pathological diagnosis was extrathoracic tissue in most cases, which lacks pathological records related to the lung and only meets criteria such as specific swelling/mass and elevated serum IgG4 levels. Therefore, the diagnosis of IgG4-RLD may be overestimated based on symptoms and imaging findings, particularly in patients who do not show improvement after treatment. Third, owing to the lack of lung function test results, it was impossible to evaluate the impact of diseases on lung function. Finally, because the inclusion criteria required an ACR/EULAR score of >20, patients with solitary lung involvement were excluded.

## Conclusion

Chest lesions in IgG4-RD, particularly in patients with lung involvement, can exhibit various abnormalities that are often ignored. Peribronchovascular involvement and lymph node enlargement are the most common manifestations. Nodules, interstitial changes, ground-glass opacities, pleural disease, and mediastinal fibrosis are rare, and their symptoms are nonspecific. Chest CT scans should be suggested to be perform when ocular symptoms, pancreatitis, pituitary adenitis, Takayasu arteritis, or abnormally elevated IgG4 levels (>135 mg/dL) are present, and IgG4-RLD should be considered.

## Data Availability

Upon reasonable request, the corresponding author can provide data to support the findings of this study. This work is based on a pre-print version of the article available at https://doi.org/10.21203/rs.3.rs-3812318/v1.

## References

[1] Duvic C, Desrame J, Lévêque C, et al. Retroperitoneal fibrosis, sclerosing pancreatitis and bronchiolitis obliterans with organizing pneumonia. Nephrol Dial Transplant. 2004;19(9):2397–2399. doi:10.1093/ndt/gfh050. 15299101

[2] Fei Y, Shi J, Lin W, et al. Intrathoracic involvement in Immunoglobulin G4-Related Sclerosing Disease. Medicine. 2015;94(50):e2150. doi:10.1097/MD.0000000000002150. 26683924 PMC5058896

[3] Corcoran JP, Culver EL, Anstey RM, et al. Thoracic involvement in IgG4-related disease in a UK-based patient cohort. Respir Med. 2017;132:117–121. doi:10.1016/j.rmed.2017.10.005. 29229083

[4] Morales AT, Cignarella AG, Jabeen IS, et al. Updates on IgG4-related lung disease. Eur J Intern Med. 2019;66:18–24. doi:10.1016/j.ejim.2019.06.010. 31227290

[5] Zen Y, Kitagawa S, Minato H, et al. IgG4-positive plasma cells in inflammatory pseudotumor (plasma cell granuloma) of the lung. Hum Pathol. 2005;36(7):710–717. doi:10.1016/j.humpath.2005.05.011. 16084938

[6] Zen Y, Inoue D, Kitao A, et al. IgG4-related lung and pleural disease:a clinicopathological study of 21 cases. Am J Surg Pathol. 2009;33(12):1886–1893. doi:10.1097/PAS.0b013e3181bd535b. 19898222

[7] Stone JH, Khosroshahi A, Deshpande V, et al. IgG4-related disease: recommendations for the nomenclature of this condition and its individual organ system manifestations. Arthritis Rheum. 2012;64(10):3061–3067. doi:10.1002/art.34593. 22736240 PMC5963880

[8] Wallace ZS, Naden RP, Chari S, et al. 2019 American College of Rheumatology/European League Against Rheumatism classification criteria for IgG4-related diseases. Ann Rheum Dis. 2020;79(1):77–87. doi:10.1002/art.41120. 31796497

[9] Umehara H, Okazaki K, Masaki Y, et al. Comprehensive diagnostic criteria for IgG4-related disease (IgG4-RD), 2011. Mod Rheumatol. 2012;22(1):21–30. doi:10.1007/s10165-011-0571-z. 22218969

[10] Matsui S, Yamamoto H, Minamoto S, et al. Proposed diagnostic criteria for IgG4-related respiratory diseases. Respir Investig. 2016;54(2):130–132. doi:10.1016/j.resinv.2015.09.002. 26879484

[11] Matsui S. IgG4-related respiratory disease. Mod Rheumatol. 2019 Mar;29(2):251-256.; Epubicahead of print. doi:10.1080/14397595.2018.1548089. 30474465

[12] Sun X, Liu H, Feng R, et al. Biopsy-proven lgG4-related lung disease. BMC Pulm Med. 2016;16(20):20. doi:10.1186/s12890-016-0181-9. 26809651 PMC4727342

[13] Umehara H, Okazaki K, Nakamura T, et al. Current approach to the diagnosis of IgG4-related disease: a combination of comprehensive diagnostic and organ-specific criteria. Mod Rheumatol. 2017;27(3):381–391. doi:10.1080/14397595.2017.1290911. 28165852

[14] Romain M, Paul H, Mikael E, et al. Thoracic involvement and imaging patterns in IgG4-related diseases. Eur Respir Rev. 2021;30:210078. doi:10.1183/16000617.0078-2021. 34615698 PMC9488667

[15] Zachary SW, Cory P, Mark M, et al. IgG4-related disease. Clin Chest Med. 2019;40(3):583–597. doi:10.1016/j.ccm.2019.05.005. 31376893 PMC7133392

[16] Ryu JH, Yi ES. Immunoglobulin G4-related disease and the lung. Clin Chest Med. 2016;37(3):569–578. doi:10.1016/j.ccm.2016.04.017. 27514601

[17] Liu J, Liu Y, Shen X, et al. Clinicopathological characteristics of IgG4-related lung disease. BMC Pulm Med. 2021;21(1):413. doi:10.1186/s12890-021-01781-3. 34911521 PMC8672518

[18] Lv X, Gao F, Liu Q, et al. Clinical and Pathological characteristics of IgG4-related interstitial lung disease. Exp Ther Med. 2018;15(2):1465–1473. doi:10.3892/etm.2017.5554. 29434730 PMC5776625

[19] Perelas A, Silver RM, Andrea VA, et al. Systemic sclerosis-associated interstitial lung disease. Lancet Respir Med. 2020;8(3):304–320. doi:10.1016/S2213-2600(19)30480-1. 32113575

[20] Yan X, Anji X, Marion T, et al. Lung nodules and IgG4 related disease: a single-center based experience. BMC Pulm Med. 2020;20(1):218. doi:10.1186/s12890-020-01250-3. 32795329 PMC7427868

[21] Ahuja J, Arora D, Kanne JP, et al. Imaging of the pulmonary manifestations of connective tissue disease. Radiol Clin North Am. 2016;54(6):1015–1031. doi:10.1016/j.rcl.2016.05.005. 27719973

[22] Bledsoe JR, Wallace ZS, Deshpande V, et al. Atypical IgG4+ plasmacytic proliferation and lymphomas: characterization of 11 cases. Am J Clin Pathol. 2017;148(3):215–235. doi:10.1093/AJCP/AQX067. 28821195

[23] Kiil K, Bein J, Schuhmacher B, et al. A high number of IgG4-positive plasma cells ruled out nodular lymphocyte-predominant Hodgkin’s lymphoma. Virchows Arch. 2018;473(6):759–764. doi:10.1007/s00428-018-2460-8. 30259184

[24] Mirmomen SM, Sirajuddin A, Nikpanah M, et al. Thoracic involvement in Erdheim-Chester disease: computed tomography imaging findings and their association with the BRAFV600E mutation. Eur Radiol. 2018;28(11):4635–4642. doi:10.1007/s00330-018-5421-3. 29736852

[25] Surabhi VR, Chua S Rajan PP, Naoki T, et al. Inflammatory myofibroblastic tumors: current update. Radiol Clin North Am. 2016;54(3):553–563. doi:10.1016/j.rcl.2015.12.005. 27153788

[26] Matsui S. IgG4-related respiratory diseases. Mod Rheumatol. 2019;29(2):251–256. doi:10.1080/14397595.2018.1548089. 30474465

